# Statistical methods for detecting periodic fragments in DNA sequence data

**DOI:** 10.1186/1745-6150-6-21

**Published:** 2011-04-28

**Authors:** Julien Epps, Hua Ying, Gavin A Huttley

**Affiliations:** 1 School of Electrical Engineering and Telecommunications, The University of New South Wales, Sydney, NSW 2052, Australia; 2 John Curtin School of Medical Research, Australian National University, Canberra, ACT 0200, Australia; 3 Plant Industry, CSIRO, Canberra, ACT 0200, Australia

## Abstract

**Background:**

Period 10 dinucleotides are structurally and functionally validated factors that influence the ability of DNA to form nucleosomes, histone core octamers. Robust identification of periodic signals in DNA sequences is therefore required to understand nucleosome organisation in genomes. While various techniques for identifying periodic components in genomic sequences have been proposed or adopted, the requirements for such techniques have not been considered in detail and confirmatory testing for a priori specified periods has not been developed.

**Results:**

We compared the estimation accuracy and suitability for confirmatory testing of autocorrelation, discrete Fourier transform (DFT), integer period discrete Fourier transform (IPDFT) and a previously proposed Hybrid measure. A number of different statistical significance procedures were evaluated but a blockwise bootstrap proved superior. When applied to synthetic data whose period-10 signal had been eroded, or for which the signal was approximately period-10, the Hybrid technique exhibited superior properties during exploratory period estimation. In contrast, confirmatory testing using the blockwise bootstrap procedure identified IPDFT as having the greatest statistical power. These properties were validated on yeast sequences defined from a ChIP-chip study where the Hybrid metric confirmed the expected dominance of period-10 in nucleosome associated DNA but IPDFT identified more significant occurrences of period-10. Application to the whole genomes of yeast and mouse identified ~ 21% and ~ 19% respectively of these genomes as spanned by period-10 nucleosome positioning sequences (NPS).

**Conclusions:**

For estimating the dominant period, we find the Hybrid period estimation method empirically to be the most effective for both eroded and approximate periodicity. The blockwise bootstrap was found to be effective as a significance measure, performing particularly well in the problem of period detection in the presence of eroded periodicity. The autocorrelation method was identified as poorly suited for use with the blockwise bootstrap. Application of our methods to the genomes of two model organisms revealed a striking proportion of the yeast and mouse genomes are spanned by NPS. Despite their markedly different sizes, roughly equivalent proportions (19-21%) of the genomes lie within period-10 spans of the NPS dinucleotides {*AA, TT, TA*}. The biological significance of these regions remains to be demonstrated. To facilitate this, the genomic coordinates are available as Additional files 1, 2, and 3 in a format suitable for visualisation as tracks on popular genome browsers.

**Reviewers:**

This article was reviewed by Prof Tomas Radivoyevitch, Dr Vsevolod Makeev (nominated by Dr Mikhail Gelfand), and Dr Rob D Knight.

## Background

Strong functional evidence indicates that periodic dinucleotides contribute to the positioning of nucleosomes, establishing the need for robust identification of periodic signals in DNA sequences as a prerequisite to understanding nucleosome organisation in genomes. Nucleosomes are the fundamental packaging unit of eukaryote DNA and serve a critical function in the epigenetic control of gene regulation. Consisting of ~ 146 bp of DNA wrapped around a histone octamer, nucleosomes affect the accessibility of DNA to the gene regulatory apparatus. A role for DNA sequence in influencing nucleosome locations has been conjectured for some time [1] with a ~ 10 bp periodicity of certain dinucleotides being identified as a nucleosome positioning sequence ([2], hereafter NPS). The functional significance of putative NPS has been convincingly demonstrated in a number of independent studies [3-7]. Recent technological advances have enabled genome wide mapping of nucleosome locations, generating substantial interest in understanding the role of DNA sequence in their location.

A renewed appreciation for the biological significance of periodic dinucleotides in encoding nucleosome positions has been stimulated by studies that employ ChIP-chip or ChIP-seq technologies. A role for periodic elements in nucleosome positions is supported by numerous observations including: the enrichment for ApA/TpT/TpA dinucleotides at 10 bp intervals in chicken DNA [2] with an accompanying counter-phase oscillation of the GpC dinucleotide; comparable enrichments in yeast nucleosomal DNA [3] and sequences from SELEX experiments [6]; and mutagenesis experiments where single nucleotide changes substantially diminished the affinity of DNA for binding the histone octamer [6]. The mechanistic basis for the existence of these signals is putatively their influence enabling bending of DNA around the histones [8,9]. A ~10 bp period in motifs that facilitate bending is expected given this is the span of a single turn of the double helix. The genomic occurrence of period-10 elements remains unclear.

**Figure 1:**
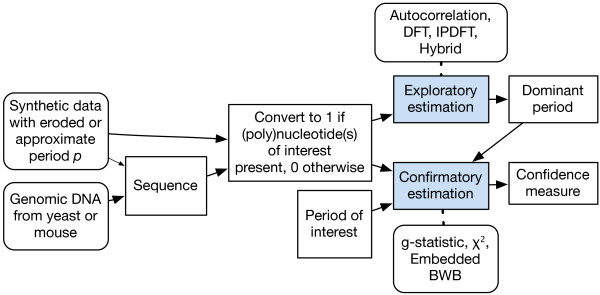
**Overview of methods for estimating periodic signals from sequence data**. In this work, both synthetic and real data are employed after a symbolic to numeric conversion. The smaller arrow connecting Synthetic data with Sequence represents a possible connection but in this study we directly synthesized the numeric data. The methods applied to exploratory or confirmatory period estimation are indicated above these elements. The embedded blockwise bootstrap (BWB) methods are embedded Autocorrelation, embedded Hybrid and embedded integer period discrete Fourier transform (IPDFT).

To date, while various techniques for identifying periodic components in genomic sequences have been proposed or adopted, the requirements for such techniques have not been considered in detail. Most studies employ period estimation techniques explicitly or implicitly for one of two purposes: exploratory or confirmatory. The conceptual relationship between these techniques and the methodological approach taken in this work is shown in Figure 1. Exploratory period estimation seeks to *discover *the existence of dominant periodic components in a sequence. Confirmatory period estimation seeks to detect the strength of a given (e.g. putatively dominant) periodic component and determine its significance relative to the remaining sequence components (see for example the analysis in Table 1 below). Confirmatory period estimation can be used in an exploratory manner (e.g. by exhaustive testing of relevant period candidates), however using exploratory techniques in a confirmatory manner may lead to erroneous attribution of significance to a particular periodic component [10,11].

**Table 1:** Significant period-10 sequences in WP nucleosomes for embedded Autocorrelation, embedded IPDFT and embedded Hybrid.

Period	**Autocorr**.		IPDFT		Hybrid	
	
	%	Total	%	Total	%	Total
2	0.03	2895	3.12	448	1.91	471
3	0.00	2910	1.62	802	0.40	995
4	0.00	2414	1.32	531	0.38	792
5	0.00	2352	2.29	830	1.71	1172
6	0.00	2389	2.20	954	0.29	1375
7	0.00	1629	1.21	988	0.08	1209
8	0.07	1493	1.62	1048	0.23	1298
9	0.12	1707	1.17	1026	0.88	1371
**10**	**3.78**	**1428**	**69.0**	**1304**	**44.07**	**1586**
11	0.00	1240	1.05	1234	1.05	1426
12	0.07	1385	1.37	1244	0.77	1434
13	0.00	1030	1.50	1336	0.43	1406
14	0.00	939	1.66	1147	0.00	1230
15	0.00	836	1.34	1042	0.29	1036
16	0.00	625	1.04	1058	0.20	995
17	0.00	591	2.09	910	0.00	870
18	0.00	605	1.68	951	0.32	943
19	0.00	550	1.81	720	0.27	748
20	0.00	419	2.10	1002	0.94	853

A sequence was counted as significant if *P_BWB_*(period-10) < 0.05. Period - dominant period for the sequence; Total - the number of sequences identified with that dominant period; % the percentage of Total sequences with the period for which *P_BWB_*(period-10) < 0.05.

The most commonly used examples of exploratory period estimation are autocorrelation (e.g. [12-16]) and the Fourier transform (e.g. [2,17-19]). Correlation based methods have been applied widely in sequence analysis, are attractive since they operate directly on the symbolic sequence, are equally spaced in period (referred to here as 'linear-period') and are relatively tolerant of both eroded perfect and approximate periodicity. Unfortunately, autocorrelation suffers from multiple-period errors, since a perfectly *p*-periodic symbolic sequence looks essentially identical at autocorrelation lags of *p*, 2*p*, 3*p*, etc [11], and from suppression of longer periods by shorter periods [10,11,20].

Fourier-based methods are well established but their spectra are equally spaced in frequency rather than in period and require a suitable symbolic-to-numeric mapping [18]. Perfectly *p*-periodic symbolic sequences exhibit a discrete Fourier transform (DFT) magnitude spectrum (of the indicator sequence for the periodic symbol) with equal intensity peaks at frequencies 2*πk*/*p*, *k *= 0, 1,..., *p *- 1, which often cause period sub-multiple errors, i.e. *p*/*k*, *k *> 1, during period estimation. One approach that is more suitable in terms of frequency spacing is the integer period DFT [21], and while this is able to identify the period expected from the NPS on a linear-period scale it too has problems. Like the DFT, it suffers from period sub-multiple errors, due to the spectral harmonics that are intrinsic to a sinusoidal interpretation of periodicity applied to the numerical mappings of symbolic periodic signals [11].

Alternative exploratory approaches that have not, to our knowledge, been applied to biological problems include the periodicity transform [22] and the exactly periodic signal decomposition [23], which are linear in period. Another example is maximum likelihood period estimation [24], which has been shown to perform well for eroded sequences, and accounts for several key types of periodicity. Dyadic wavelet methods, notably including use of the Haar basis, are of interest as an orthogonal decomposition [25,26], however these can only be applicable to exponential period scales, e.g. periods 2*^r^, r *∈ ℵ. In general, exploratory period estimation methods suffer from the lack of an orthogonal integer-periodic signal decomposition [22]. A key desirable property of confirmatory period estimation is a measure of statistical significance.

Biological sequences are rarely exactly periodic and the estimated period returned by exploratory analysis may be anywhere from very weakly to very strongly dominant with respect to the remaining sequence components (which may contain other periods or be essentially non-periodic). A measure of significance facilitates comparison with, for example, other candidate periods of interest or the strength of the same periodic component in other sequence data. In practise, period estimation techniques have been widely applied in the genomics literature with little or no consideration of the statistical significance of the period estimate.

Examples of confirmatory period estimation include quantifying the significance of Fourier-based period estimates via the exponential distribution of Fourier magnitudes [27] and its extension to the significance of multi-harmonic peaks in symbolic sequences using extreme value statistics [28]. These examples are specific to Fourier techniques and do not appear to have been widely adopted. Another example is significance measures derived empirically by repeated random selection of fixed-length sequence fragments followed by signal to noise ratio calculations [29]. Chaley et al. [30] applied a mutual information criterion to explore latent periodicity in sequence data using Z-scores based on mutual information. Although this approach can be used for confirmatory purposes, their application was essentially an exploratory one. In problems where the strength of periodicity is of interest, resampling methods are applicable. In these methods, the strength of the maximum likelihood period estimate is measured relative to the same period estimate derived from randomly resampled instances of the same sequence. Permutation tests are a special case of resampling methods, in which resampled blocks have a length of just one, and for which resampling is performed without replacement. In the permutation method employed by Ahdesmaki et al. [31], time series data are randomly permuted to estimate a distribution and hence *p*-value for the periodicity strength. However, as noted by Ptitsyn et al. [32], this method allows random re-institution of periodicity unless permutations in position of size *p *are avoided. In their method, developed for short microarray time series, Ptitsyn et al. [32] count the number of permutations (with period-*p *deliberately avoided) whose periodogram peak at *p *is larger than that of the time series under test. More recently Ying et al. [33] employed a blockwise resampling approach on sequence data. Resampling at the level of single units is not valid for biological sequence data as nucleotides do not occur randomly. This non-randomness is evident in the triplet nature of the genetic code and, in non-protein coding DNA sequences, the non-random occurrence of dinucleotides. Of particular significance here is the widespread deficit of the dinucleotide TA [34], a component of the NPS. Such non-randomness in the observed sequences compared with those generated by permutation test approaches will cause the presumed Type I or II error rates (depending on whether the signature dinucleotides occur less or more frequently than expected given their component nucleotide frequencies) to be incorrect and thus lead to incorrect inferences. Further confounding the problem are larger scale correlations in sequence composition ([35], for example).

Here we evaluate the suitability of selected exploratory signal processing techniques and confirmatory statistical techniques for estimation of dinucleotide periods pertinent to the nucleosome positioning problem, initially via simulation. We then apply these results to assess whether we detect the reported 10 bp period for the dinucleotides {*AA, TT, TA*} in yeast and assess the relationship between this evidence and the functional classification of the nucleosomes. Finally, we search the genomes of yeast and mouse for regions with statistically significant 10 bp periods.

## Results and Discussion

Figure 1 presents an overview of the process by which estimates of the periodicity of symbolic data were obtained in this study. Irrespective of whether the starting material was biological or synthetic, all subsequent steps in the analyses are common. As indicated in the figure, the different period metrics we employed (defined in the methods) were applied to both the exploratory and confirmatory approaches.

### Methods for detecting periodic fragments

#### Numerical Representation and Periodicity

In this investigation, we represent DNA sequence fragments *s*[*n*], where *n *∈ ℤ is a position index, using the binary indicator sequence numerical representation [36]. That is,(1)

This representation is commonly employed and can be simply generalised to produce several other existing numerical representations. For the purposes of this paper we define perfect *p*-periodicity in a fragment of length *N *as(2)

where *n*_0 _is the position of the first dinucleotide(s) of interest and *δ*[*n*] is the Kronecker delta. In practise perfect periodicity is very rare, and hence we also define (i) imperfect or eroded periodicity [30], which contains substitutions of the dinucleotide(s) of interest, and (ii) approximate periodicity, in which the period changes randomly from one cycle to the next but has expected value *p*, representing insertions or deletions of the dinucleotide(s) of interest.

#### Significance Measures for Confirmatory Period Estimation

In this study, we employ four period estimation techniques, derived from the two most commonly employed groups of techniques in the literature: autocorrelation and Fourier-based methods. The integer period discrete Fourier transform (IPDFT) is a variant of the well-known discrete Fourier transform (DFT) that is tailored for the integer-period problem of exploratory period estimation, however the two are virtually equivalent for the purposes of confirmatory period estimation. The Hybrid autocorrelation-IPDFT combines the strengths of the autocorrelation and DFT methods for exploratory period estimation.

Four measures of periodicity significance are considered: Two are based on the *g*-statistic and resampling (blockwise bootstrap (BWB)) approaches discussed in the background, while the Chi-squared measure is new in the current context. The Cramér-Rao bound (CRB) developed herein is applicable to Fourier-based period estimation, and although it is not strictly a test, it gives a measure of the strength of periodicity. To distinguish between exploratory period estimation and BWB based on a particular period estimation technique, we will refer to the use of period estimation methods within BWB as embedded. For instance, a BWB using the IPDFT to determine the peak values |*S*[*p*]| of resampled sequence fragments will be referred to as employing an embedded IPDFT. Formal definitions and full details of all measures are given in the Methods section.

### Statistical properties of period estimation (from simulated data)

We evaluated the statistical properties of the different techniques by first applying them to simulated data. The striking dinucleotide periodicities reported for nucleosome associated DNA are typically presented as means of many sequences. Individual sequences are unlikely to perfectly match this signature due to the effect of mutation. We considered two different definitions of imperfectly periodic sequences: (i) imperfectly periodic due to *erosion *of a perfectly period-10 signal; (ii) imperfectly periodic due to the underlying signal being *approximately *period-10. We simulated eroded sequences by generating 100 synthetic perfectly period-10 symbolic sequences of length *N *= 150 and then eroding these in increments of 1% to a maximum of 50% erosion. We simulated approximately periodic symbolic sequences using a Gaussian distributed period with expected value of 10 and standard deviation *σ*. These were then repeated for many standard deviations *σ *∈ [0.1, 3].

**Figure 2:**
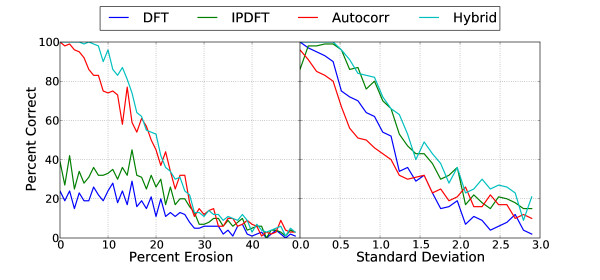
**Average accuracy of dominant period estimation for simulated sequences**. Eroded (left) - sequences were period-10 synthetic sequences of length *N *= 150, eroded to the extent indicated on the x-axis. Approximate (right) - sequences were of length *N *= 150, with periods Gaussian distributed about an expected value of 10 bp.

Results of the period estimation comparison are shown in Figure 2. In this experiment, a sequence length of *N *= 150 was selected, this being approximately the length of nucleosome associated DNA. If *N *is varied from 150, e.g. to 149, the advantage of the IPDFT over the DFT is not preserved, as is also noted in Epps [11], however for the purposes of this paper the IPDFT can be considered a better choice since the periods it evaluates are not dependent on the sequence fragment length, while the reverse is true for the DFT. The relative advantage of the autocorrelation over the IPDFT is also not necessarily preserved when *N *is varied, in particular for prime values of *N *[11]. The greater accuracy in estimation of the dominant period by the Hybrid derives from the complementary nature of the flaws in the Fourier transform and autocorrelation measurements.

When synthetic period-10 sequences are degraded instead by varying the period to produce approximate periodicity, and a similar analysis to that for Figure 2 performed, Fourier-based methods appear to generally outperform autocorrelation. This can be explained by the fact that the period was allowed to vary within each sequence fragment but with a mean of 10 bp, so that Fourier methods with a fixed 10 bp period basis function are better suited to the problem than autocorrelation, which depends on exact similarity between *s*[*n*] and *s*[*n *+ 10] for the *n*'th sequence position index. Comparing the two panels in Figure 2, it can be noted that the Hybrid approach generally retains the better period estimate from either the autocorrelation or Fourier-based methods in each case. Reasons for this were discussed in Epps [11]. To summarise the comparison of exploratory period estimation methods for synthetic data, results show that IPDFT and Hybrid were roughly equivalent in their average accuracy for approximately periodic sequences, while the Hybrid was considerably superior on eroded synthetic sequences (Figure 2).

**Figure 3:**
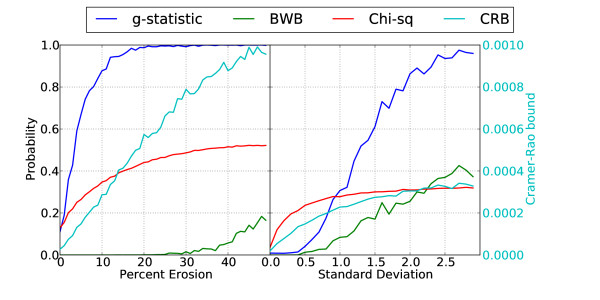
**Significance measures from the embedded IPDFT (g-statistic and BWB) for period-10 synthetic sequences of length *N* = 150**. Sequences were either erosion of a perfect period-10 signal (top) or had periods Gaussian distributed about an expected value of period-10 (bottom).

The results of the significance measure comparison for imperfect and approximate periodicities are seen in Figure 3, for fragment lengths *N *= 150 each expressed in terms of the probability of an equivalent or larger periodic component at exactly period-10 (for the BWB and Chi-squared measures) or the probability of an equivalent or larger peak in the periodicity profile |*X_IP_*[*p*]| (for the *g*-statistic). All measures show the expected increasing probability with degradation to the sequence fragment periodicity, and all are monotonic, although the Chi-squared measure is the smoothest for these simulations.

The preceding experiments yield understanding of the comparative behaviour of exploratory and confirmatory approaches to period estimation as a function of the degree of periodicity in the underlying sequence. As discussed in the introduction, an important characteristic of confirmatory approaches is the extent to which they are able to detect the presence of periodicity that may be degraded in one way or another. Since the problem of detection is generally dependent on the detection threshold chosen for a specific application, a conventional method of comparison is to calculate the receiver operator characteristic (ROC) curve (true positives vs. false positives) for each method.

**Figure 4:**
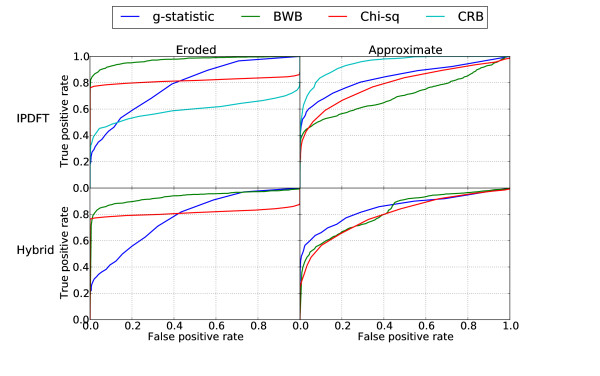
**ROC curves for confirmatory period detection of eroded (left) and approximate (right) synthetic period-10 sequences and randomly permuted sequences for embedded IPDFT (top) and embedded Hybrid (bottom)**.

The results of the ROC simulation for eroded and approximately periodic sequence fragments can be seen in Figure 4. The results for approximately periodic sequences, shown for period-10 only, are slightly simplistic, since if it is known or suspected a priori that the period-10 component may be approximate rather than exact, a more reasonable approach may be to also consider the strength of components with periods 9, 11 etc. Under these circumstances, the order of preference among the various techniques compared herein may differ from that suggested by the results in Figure 4. Interestingly, between the two panels in Figure 4, the order of effectiveness of the various methods is reversed. Since the difference between the significance measures is most marked for Figure 3, where the BWB performs close to the ideal behaviour for a period-10 detector, we selected the BWB for further experimental work. An explanation for the good performance of the CRB for approximate periodicity can be constructed along similar lines to that for Fourier-based methods in the periodicity degradation simulations above, since CRB is also Fourier-based. Although the CRB is very effective for approximate periodicity, which occurs commonly in practise, the simulation constraint of an average period of 10 bp is artificial and probably overstates the practical utility of the measure somewhat.

### Analysis of Yeast Chip-chip data

The yeast ChIP-chip data identified 73327 DNA sequences that are associated with nucleosomes in vivo versus their linker sequences. Lee et al. [37] further utilised the variance in association to classify the sequences into 31557 well-positioned or 41770 fuzzy nucleosomes. Since the regions identified by Lee et al differed in length, and statistical power of the period estimation methods are sensitive to length, we modified their sequence coordinates such that sequence fragments from each class were all exactly 150 bp long. Only sequence coordinates that were independent (did not overlap with any other coordinates) were used. See Methods for detailed description of the sampling process.

**Figure 5:**
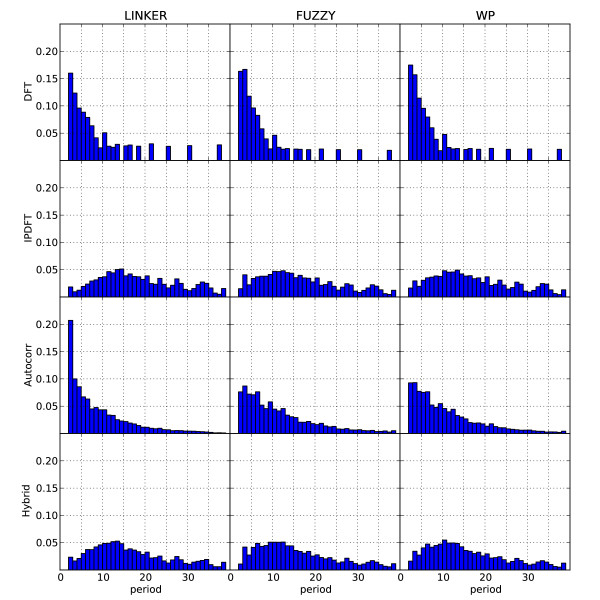
**Dominant period histograms for well-positioned (WP) yeast nucleosome DNA sequence fragments, estimated using DFT, IPDFT autocorrelation and Hybrid autocorrelation-IPDFT**.

In Figure 5 we plot the distribution of dominant periods from application of the different metrics to the different sequence classes. Note that these distributions are not periodicity profiles typical of exploratory period estimation applied to a single sequence fragment, but the aggregation of dominant period counts derived from exploratory period estimation over many sequence fragments. Neither the DFT nor autocorrelation approaches identify the period-10 mode expected (at least for the well-positioned (WP) sequence class). The IPDFT identifies a mode of ~11-12 bp for both nucleosome associated classes whilst the Hybrid metric exhibits a mode at period-10 for well-positioned sequences and a highly similar result for fuzzy nucleosome associated sequences. In contrast, the location of the distribution estimated from linker sequences is right-shifted by ~5-bp for both the IPDFT and Hybrid statistics.

Comparison of the dominant period estimation using the BWB results suggests that the embedded IPDFT has greater sensitivity for identifying significant period-10 signals (Table 1). For each sequence class, for each integer period, we evaluated the fraction of sequences that returned a nominally significant BWB test probability (*P_BWB_*(period-10) < 0.05). In Table 1 each row represents the results for well-positioned sequences identified as having a dominant period at the indicated value. For instance, of the 2895 sequences with a dominant period of 2 according to the autocorrelation statistic, only 1 had a *P_BWB_*(period - 10) < 0.05. The results indicate that while more sequences were identified with a dominant signal at period-10 using the Hybrid metric during exploratory period estimation, ~30% more sequences were identified with nominally significant period-10 using embedded IPDFT during confirmatory analysis. For sequences with dominant period not equal to period-10, the proportion identified as having significant *P_BWB_*(period-10) was ~10-fold higher for embedded IPDFT compared with embedded Hybrid, but still typically less than 2%. In contrast to these results, the embedded autocorrelation statistic did extremely poorly in identifying period-10 sequences, failing to register a proportion greater than 5%. It is also noteworthy that for all embedded (confirmatory) period estimation methods shown in Table 1 the BWB does not return a large proportion of significant sequences for periods 2, 5 or 20, strongly suggesting that the BWB appears to be robust to the known confounders of factor and multiple periods during confirmatory analysis.

To summarise the exploratory testing, only the distribution of Hybrid estimates appear to identify period-10 as the mode for both Fuzzy and well-positioned nucleosome sequences, although IPDFT is close to this (Figure 5 and Table 1). The distribution from IPDFT was slightly inflated relative to Hybrid, with the mode at 13. The deflation of the Hybrid estimates relative to those of the IPDFT is due to the autocorrelation factor in the Hybrid formulation, which drops off with increasing period, due to the finite window length of the sample autocorrelation.

For confirmatory testing, the relative performance advantage of IPDFT and Hybrid is reversed. A substantially larger fraction of windows have nominally significant embedded IPDFT compared with embedded Hybrid (Table 1). The demonstrated sensitivity of IPDFT to detecting sub-multiple periods [11] is only weakly evident, with modest increases in the percentage of windows significant at the sub-multiple periods (2 and 5). These increases were readily distinguished from the ~70% of windows with dominant period-10 significant. In contrast, less than half of windows with dominant signal at period-10 were significant using the embedded Hybrid. The trend in these different sensitivities from the analysis of yeast data was also evident in the ROC curves (Figure 4), albeit less pronounced. In striking contrast to the performance of IPDFT and Hybrid, embedded autocorrelation did extremely poorly and quite clearly should not be used for confirmatory analysis.

### Analysis of entire genomes

We evaluated the proportion of the yeast and mouse genomes containing the NPS. All possible 150 bp windows in each genome were evaluated and estimation of *P_BWB _*restricted to windows for which the dominant period was 10. The BWB used *R *= 250 resamplings with the embedded IPDFT metric. In our analysis of the complete genome of yeast, 484436 windows were identified with dominant period equal to 10. After merging overlapping period-10 windows, there were 15457 segments, 12468 of which were defined by windows with *P_BWB _*< 0.05. The combined length of segments with significant period-10 was 2557556 bp which is ~21.2% of the entire yeast genome.

An equivalent analysis applied to the mouse genome revealed a comparable abundance of NPS sequences. A total of 82583288 windows were identified with dominant period 10 and merging of overlapping windows indicates ~23% of the mouse genome is spanned by NPS. When only windows with *P_BWB _*< 0.05 (using embedded IPDFT) were considered, these reduced to 2465525 total windows covering ~18.9% of the haploid mouse genome.

## Conclusions

This paper has compared four period estimation methods and four significance measures suitable for the problem of symbolic period detection via simulation, and evaluated the suitability of these for estimation of dinucleotide periods pertinent to the nucleosome positioning problem. For estimating the dominant period, we find the Hybrid period estimation method empirically to be the most effective for both eroded and approximate periodicity, combining the strengths of autocorrelation and the IPDFT in the two types of imperfect periodicity respectively. The BWB was found to be effective as a significance measure, performing particularly well in the problem of period detection in the presence of eroded periodicity. When applying confirmatory analysis of the period-10 component using the BWB on yeast ChIP-chip data, where a strong 10 bp {*AA, TT, TA*} dinucleotide periodicity is expected for well-positioned and fuzzy labelled sequence fragments, two key insights emerged: significance testing using the BWB appears to be robust to the known confounders of factor and multiple periods, regardless of the period estimation technique; and the autocorrelation method is poorly suited for use with the BWB, detecting only a small fraction of the expected sequence fragments containing significant 10 bp periodicity. During simulations, the Hybrid method was found to be outperformed by the IPDFT in detecting significant 10 bp periodicity when the periodicity is eroded but outperformed IPDFT when the periodicity was approximate. While the BWB is computationally intensive, we successfully applied it to a large vertebrate genome. This was facilitated by only computing the probability for windows where the dominant period matched our prior hypothesis. Reducing the number of resamplings or increasing the spacing between windows can also be employed to allow a fuller inspection of the genome.

Application of our methods to the genomes of two model organisms revealed a striking proportion of the yeast and mouse genomes are spanned by NPS. Despite their markedly different sizes, roughly equivalent proportions (19-21%) of the genomes lie within period-10 spans of the NPS dinucleotides {*AA, TT, TA*}. The biological significance of these regions remains to be demonstrated. To facilitate this, the genomic coordinates are available as Additional files 1, 2 and 3 in a format suitable for visualisation as tracks on popular genome browsers.

The utility of methods developed in this work are not restricted to the problem of detecting NPS. For instance, confirmatory testing could also be applied to detection of protein coding sequences in unannotated genomes by evaluating period-3 occurrence of nucleotides. Importantly, given the intrinsically periodic structure of DNA it seems plausible that information encoding elements other than NPS may also be influenced by this organisation. The work presented herein on both exploratory and confirmatory analyses provide guidelines for identifying the occurrence of periodic elements and thus advancing our understanding of their role in genome organisation.

## Methods

### Exploratory Period Estimation

The discrete Fourier transform (DFT) is defined as(3)

where *N *is the length of the DNA sequence fragment, and *k *∈ ℕ, is a frequency index corresponding to a period *p *= *N*/*k*. The DFT is thus only evaluated at selected integer periods, whose values depend on *N*, while typically integer periods are of most interest. For this reason, we also consider a second technique, the integer period discrete Fourier transform (IPDFT) [21], which is a variant of the DFT evaluated only at integer periods:(4)

where *p_max _*is the longest period of interest. It should be noted that the Fourier components of the IPDFT are not all mutually orthogonal. For Fourier-based methods, explicit bounds on the period resolution can be determined. For example, in sequence fragment lengths of 150 bp (rounding up the length of nucleosome associated DNA), the period beyond which adjacent integer-spaced periods cannot be resolved using Fourier-based methods (with a rectangular window) is approximately . Thus period-10 behaviour can be adequately estimated by these methods, to the resolution of the nearest integer period. The autocorrelation is widely used in period estimation for sequence data, and is defined as:(5)

Finally, we also use the recently developed Hybrid autocorrelation-IPDFT technique, which has shown promise for overcoming some of the limitations of the DFT and autocorrelation for period estimation in sequence data [11].(6)

In each case, the period estimate , where *S*[*p*] ∈ {*X*[*p*], *X_IP _*[*p*], *r_xx_*[*p*], *H_x_*[*p*]}, is taken to be the dominant periodicity of the sequence, and which we refer to as the dominant period throughout this work.

### Significance Measures for Confirmatory Period Estimation

#### Fourier-Based Period Estimation Variance Bounds

Fourier methods are often used for maximum-likelihood estimation of a dominant frequency (period) in a numerical signal, both within [18,19] and outside [38] the genomics literature. For this estimate, it is possible to derive a Cramér-Rao bound (CRB) on the variance of this frequency estimate [39]. Following a similar procedure, we derive a bound for the (sinusoidal) period estimate variance herein as:(7)

where  is the 'signal-to-noise ratio', which can be interpreted as the ratio of the energy of the perfectly periodic component of *x*[*n*] to that of the remaining component of *x*[*n*]. It will be noted that this bound is a very strong function of the period *p*, reflecting the period resolution effect discussed in the foregoing sub-section. This can be observed to contrast with Tretter's [39] CRB for sinusoid frequency, which is a similar function of inverted SNR but is not a function of frequency, and hence not of period. Clearly calculation of the CRB requires an estimate  of the signal-to-noise ratio, since the true 'signal' and 'noise' components are unknown. Here we use a definition based on the discrete Fourier transforms of the actual and perfectly periodic sequences (see Additional file 4) which (by Parseval's theorem) approximately preserves the conventional interpretation of the signal-to-noise ratio. The CRB is applicable to numerical signals whose periodicity is sinusoidal, or can be represented using sinusoids, and would not typically be used as a statistical test. Here, we use the CRB as a measure of the significance of a candidate period, and compare it qualitatively with the other significance measures.

#### g-Statistic

The *g*-statistic, originally proposed for the periodogram by Fisher [40], and used recently by Wichert et al. [41], Ahdesmaki et al. [31] and in a comparison by Ptitsyn et al. [32] for detecting periodicity in microarray time series data, can be generalised to the period estimation methods considered herein, in a modified form as(8)

under the assumption that the 'noise' (i.e. all signal components other than the dominant periodicity) is Gaussian. Clearly this will be a weak assumption if multiple periods are present in the signal, however in general this is not known a priori. Using the p-value based on the exact distribution of *g *[41], with notation adjusted for use herein,(9)

where *g_obs _*is the observed value of *g *and , it is possible to test whether a sequence is purely random or whether it has periodic behaviour. The *g*-statistic is applicable to numerical signals.

#### Blockwise Bootstrap

In sequence analysis, both of the permutation approaches discussed in the background section have the limitation that they disrupt the non-random background distribution of polynucleotides. In essence, neighbouring nucleotides cannot be considered to be independently distributed [34,35].

Hence, we adopt a method we refer to as the blockwise bootstrap (BWB). As in Ying et al. [33], here we resample sequences of interest by building a resampled sequence *s_p_*[*n*] from *p*-length fragments of *s*[*n*] selected at random, i.e.

(*s_p_*[*lp*], *s_p_*[*lp *+ 1],..., *s_p_*[(*l *+ 1)*p *- 1]) = (*s*[*n*], *s*[*n *+ 1],..., *s*[*n *+ *p *- 1]), *l *= 0, 1,..., ⌊*N*/*p*⌋, where *n *∈ {0, 1,..., *N *- *p*} is selected at random for each *l*. An appealing feature of this approach is that it preserves the base rate of occurrence of nucleotides (and polynucleotides up to length *p*) during the test. *R *synthetic sequences are produced by randomly resampling, with replacement, from the original sequence fragment, and the number of times *N_G _*a peak greater than or equal to  is measured at period  is recorded. Finally the *p*-value *P_BWB _*of the test sequence is determined as *N_G_*/*R*. A low *p*-value, for example less than 0.01, corresponds to fewer than 1 in 100 resampled sequences exhibiting a peak greater than or equal to  at period . Of course this test can be applied to other periods than , to ascertain the significance of a secondary peak, for example. This type of test has been applied in a very wide range of applications, e.g. [42].

Although the BWB is a fundamental test for *p*-periodicity, it has the shortcoming of being very computationally expensive to apply. Like the *g*-statistic, the significance level of the BWB is dependent on the period estimation technique, rather than merely the data and the period of interest, in contrast to the other two measures considered.

#### Pearson's Chi-Squared Test

Treating perfect periodicity as a model, whose fit to (or deviation from) the sequence data of interest is to be measured, a chi-squared test can be developed. In this case the deviation corresponds to a period estimation significance measure, while the test itself is a threshold for the measure.

To calculate the deviation, it is necessary to first define the 'model'. For a sequence fragment that is perfectly *p*-periodic with respect to symbol *s_m_*, *m *∈ {1, 2,..., *M *}, the probability mass function (pmf) is . Note that this pmf says nothing about other symbols; they could be randomly occurring or also perfectly periodic, but most often we are interested in the periodicity of a particular symbol or symbols.

Having determined the periodicity 'model', a count of the observed instances of periodicity is required. For each position in a sequence fragment *s*[*n*], note the value *s_m _*of the current symbol, look ahead by *p*, record the presence or absence of each symbol of interest, then aggregate these across all instances of *s_m _*and divide by the total number of occurrences of *s_m_*, to produce an empirical *p*-spaced pmf for each symbol.

That is, for a sequence fragment *s *comprised of symbols *s*_1_, *s*_2_,..., *s_M_*, form the set *C_m _*= {*s*[*n*] | *s*[*n*] = *s_m_*, *n *= 0, 1,..., *N *- 1} and the *M *sets , one per symbol *s_m_*. The empirical pmf for each symbol, , is then

where |*C*| denotes the number of elements in *C*.

The deviation measure can thus be constructed as

Note that the deviation measure can be quite flexibly constructed, in the sense that 'symbols' can be replaced by sets of symbols of interest, for example rather than treating AA, AT, TA separately, periodicity in any of {AA, AT, TA} can be treated as a single 'symbol'. Just as all symbols that are of interest can each be lumped together in the pmf, all symbols that are not of interest can also be lumped together, rather than listing a probability for every possible symbol (e.g.  if *s*[*n*] = *s_m _*and  if *s*[*n*] ≠ *s_m_*), which is convenient when dealing with periodic long polynucleotides. This approach has some similarities with the mutual information method for revealing latent sequence periodicity proposed by Chaley et al. [30].

Finally, from the deviation statistic a *p*-value *P_CS _*may be obtained by comparing its value with a chi-square distribution with one degree of freedom.

### Simulation of eroded and approximately periodic data

In order to compare the period estimation performance of the methods discussed above, an experiment was conducted in which 100 synthetic period-10 sequences of length *N *= 150 were generated and then eroded in increments of 1% to a maximum of 50% erosion. The erosion comprised 'flipping' the binary indicator sequence representation of equation (1) of a percentage of the *N *dinucleotides, as adopted by Arora et al. (2008) for nucleotides. This corresponds to the substitution of alternative dinucleotides for AA, TT, TA, and vice versa. The percentage of instances for which each period estimation method correctly predicted the period-10 behaviour was then recorded. This experiment was then repeated for approximate periodicity, for which we defined Gaussian distributed periods with expected value of 10 and standard deviation *σ *∈ [0.1, 3]. This corresponds to the insertion and/or deletion of non-{AA, TT, TA} dinucleotides. To better understand their properties for known periodic sequence data, all significance measures considered herein were compared on the same synthetic period-10 sequences eroded and modified to produce approximate periodicity to assess the degradation of the period estimates for non-ideally periodic sequences. In all cases the results shown are averages across 100 random degradations of a pure period-10 sequence, and the IPDFT was used for period estimation throughout, to allow comparison with the CRB (which requires a Fourier-based method). In these experiments, the BWB used *R *= 1000 random sequences.

To assess the significance measures in terms of their accuracy as confirmatory methods, a period detection simulation was constructed. For 1000 instances of a perfectly periodic synthetic sequence of length *N *= 150 with 10 bp periodicity degraded between 1% and 50% (20 instances per 1% increment), i.e. the true positives, and 1000 randomly permuted instances of the same sequence, i.e. the true negatives, the sensitivity and specificity and hence receiver operator characteristic (ROC) curves were calculated. In this experiment it is possible that a small number of highly eroded periodic sequences may strictly be true negatives and a small number of random sequences may strictly be true positives, however this is common across all methods compared. This procedure was repeated for 1000 instances of the same sequence but with the period varied randomly according to a Gaussian distribution with expected value of 10 and standard deviation *σ *∈ [0.2, 4]. (50 instances per 0.2 increment in *σ*).

### Software implementation

All methods were implemented originally in MATLAB Version 2008a. The methods for period estimation and significance testing were then independently implemented in Python and Pyrex, a language that generates C-code which is compiled into dynamically loaded Python extensions. The compiled versions substantially improve compute performance. The source code has been contributed to the open sourced genome biology toolkit PyCogent [43] and is available from the subversion repository. All genomic analyses were conducted using the Python implementation. All scripts used are available on request from the authors.

### Biological data

Yeast genome sequence coordinates for nucleosome associated DNA were obtained from Lee et al [37], whose procedure we briefly summarise. This data set was generated by analysis of a micrococcal nuclease (MNase) digestion of whole yeast genomic chromatin that had been subjected to cross-linking of histones to DNA. The resulting purified DNA fragments were then hybridized to an Affymetrix probe array with a 4 bp resolution. A Hidden Markov Model was used for detecting regions corresponding to 'well-positioned', defined as spanning 31-38 probes, or 'fuzzy', defined as spanning 39 probes, nucleosomes. Linker regions were defined as those spanning between identified nucleosome positions. Coordinates for the well-positioned, fuzzy and linker regions were downloaded from http://chemogenomics.stanford.edu/supplements/03nuc/datasets.html (dataset S5). Since these regions differed in length, and statistical power of the period estimation methods are sensitive to length, we modified these sequence coordinates such that sequence fragments from each class were all exactly 150 bp long. Specifically, the sequence coordinates from Lee et al were adjusted by equivalent symmetric expansion (in the 5' and 3' directions) until the coordinates were exactly 150 bp long. Only sequence coordinates that were independent (did not overlap with any other coordinates) were used. The genomic sequences corresponding to these coordinates were downloaded from http://www.ebi.ac.uk/~huber/yetia/yetiadata/SGD-0508/. The total number of sequences in each class were: 31557 well-positioned nucleosomes; 41770 fuzzy nucleosomes; and 10465 linker regions. Mouse genomic sequences were obtained from Ensembl release 58.

## Competing interests

The authors declare that they have no competing interests.

## Authors' contributions

JE and GAH conceived research. JE, HY and GAH contributed to study design, implementation and analysis. JE and GAH wrote the paper. All authors read and approved the final manuscript.

## Reviewers' comments

### Reviewer's report 1

Prof Tomas Radivoyevitch, Case Western Reserve University, Ohio

It would be nice if a new Figure 1 was created to give the overall organizational structure of the methods. As it stands, my default inclination is to think of the exploratory estimation methods as being "feature extractions" in pattern recognition (i.e. a data dimension reduction step). But I am not sure of this. Does any filtering happen in the frequency domain to reduce the dimensionality of the data before the statistical tests are applied? Is it correct to think of the exploratory methods as feeding into the confirmatory methods to create pipelines? If these ideas are way off, something should be stated upfront to keep readers from wandering off. Related to this, my reading first stalled on the Page 3 sentence "Confirmatory period estimation can be used in an exploratory manner (e.g. by exhaustive testing of relevant period candidates), however the reverse seems inadvisable." Here, if the exploratory approach is a preprocessing dimensionality reduction step, what is meant by the "reverse"? A new Figure 1 could nip this sort of wandering in the bud.

***Author response***: *We thank the reviewer for this suggestion and now include a *Figure 1*that describes the overall organisation of the methods, as suggested by the reviewer. All subsequent figure numbers have been incremented*.

*The perspective of period estimates as reduced-dimension 'features' and confirmatory methods as subsequent statistical tests is an interesting one, but the authors share the reviewer's reservations about its applicability here. It is not obvious to think of period estimates as having higher or lower dimensionality, except perhaps in terms of the concatenation of period estimates for different integers. In the latter case, however, because of the absence of an orthogonal set of integer-period basis functions, the 'features' must be assumed to be mutually correlated to some extent. Similarly, it is not obvious to think of confirmatory methods in the same sense as pattern recognition (even though our ROC curve analysis invites this interpretation); the objective here is rather trying to characterise the strength of a particular (e.g. putatively dominant) periodic component relative to the others*.

*No frequency domain filtering is applied; period estimation follows the usual convention of finding a peak in the periodicity profile determined using either the autocorrelation, discrete Fourier transform or Hybrid method*.

*Our comments concerning the use of exploratory period estimation for confirmatory purposes have been revised to clarify their meaning*.

### Reviewer's report 2

Dr Vsevolod Makeev, State Research Centre of Genetics and Selection of Industrial Microorganisms, GosNIIgenetika, Moscow (nominated by Dr Mikhail Gelfand)

The authors compare four methods for identification of fuzzy periodical patterns in nucleotide sequences in the context of extracting the 10-periodic nucleosome positioning signal. They compare classic approaches like autocorrelation and digital Fourier transform as well as more heuristic methods like Integer period digital Fourier transform (IPDFT) and a Hybrid method combining autocorrelation and IPDFT factors for each period. They demonstrate very convincingly, both at simulated and real nucleosome positioning data, that autocorrelation and digital Fourier transform simply cannot identify a fuzzy periodic signal. On the other hand, two heuristic measures put forward by the authors, IPDFT and the Hybrid, at least can identify the 10-period in the regions experimentally shown to contain nucleosome positioning signal (Figure 5). With the help of simulated data authors also show that the Hybrid measure is more stable for identification of the correct period from 'eroded sequences' (Figure 2). The authors also argue that the Hybrid measure gives an unbiased estimator of the dominant period, but for me this point sounds less convincing, since it is illustrated only at the real data with unreliable nucleosome positioning signal (the so called fuzzy nucleosome positioning set) and also appears less attractive from the theoretical point of view.

***Author response***: *We do not assert that the Hybrid method is unbiased, nor do we attempt to prove this or support it with empirical evidence. We further agree with the reviewer that, from a theoretical point of view, the Hybrid method has less appeal than autocorrelation or the discrete Fourier transform. Despite these issues, as our results establish, the IPDFT and Hybrid out-perform the metrics with better theoretical credentials on symbolic data*.

Indeed, in the case of fuzzy positioning the maximum for IPDFT is somewhat shifted to the left, but the distribution is very flat in this region. Moreover, the authors themselves are aware that the main reason of the deflation produced by the Hybrid estimator is the way how the autocorrelation is correlated (the number of summand depending on period *p*).

***Author response***: *This is expressed in the paper as "The deflation of the Hybrid estimates relative to those of the IPDFT is due to the autocorrelation factor in the Hybrid formulation, which drops off with increasing period, due to the finite window length of the sample autocorrelation."*

If the sequence is closed to a ring before the autocorrelation is calculated, which is more appealing from the point of view of convolution theorem, then the autocorrelation would not fade for larger periods and the deflation effect of Hybrid estimate would be much smaller (if any). From my point of view, the real strength of Hybrid estimator comes from the simulations presented in Figure 2.

***Author response***: *Cyclic autocorrelation is an interesting suggestion, and theoretically attractive as suggested. On the other hand, this implies unnatural constraints on the sequence fragment: specifically by 'closing the ring', for a sequence x*[*n*] *of length N we would be artificially connecting x*[*N - 1*] *with x*[0]. *For the signal lengths considered here, this constraint is artificial for most genomic DNA*.

I believe the work is worth publishing because the autocorrelation function and discrete Fourier transform methods have been tried countless times for identification of periodic nucleosome positioning signals in DNA sequences with a very limited success. Finally the authors demonstrate that these measures in fact perform very poorly for identification of fuzzy periodic signals in discrete data.

***Author response***: *We thank the reviewer for these incisive observations, which relate closely to the central themes of this paper. Our observation is that while the theoretical appeal and behaviour on numerical signal data has led to widespread adoption of autocorrelation and the discrete Fourier transform, for symbolic data, it is clear that they have significant limitations for some applications. These can lead to erroneous attribution (or non-attribution) of significance to a particular periodic component*.

*Makeev responds in a second review*

It is necessary to remark in the text (better in the "Methods" section, near the definition of IPDFT) that Fourier series components for IPDFT are not mutually orthogonal. The authors estimate a bound for the variance (formula 7) with the help of Parseval's theorem, but the Parseval's theorem is the consequence of orthogonality relations for Fourier coefficients. It is probably not valid for IPDFT, therefore the bound for the variance (section "Significance Measures for Confirmatory Period Estimation") is questionable in the case of IPDFT. The estimation of variance bound becomes heuristics for IPDFT.

***Author response***: *The Cramér-Rao bound itself was developed without use of Parseval's Theorem. The Fourier based signal to noise ratio calculation provided in *Additional file 4* seemed to us a more intuitive approach than one based directly on the sequence fragment. The reviewer is quite correct to point out that this calculation should only be performed using the discrete Fourier transform, and indeed it was done this way in all our experiments. In the Methods section, the introduction to the IPDFT has been extended slightly to clarify the non-orthogonality of Fourier components in the IPDFT, and the reference to *Additional file 4* now gives a brief textual overview of the signal to noise ratio calculation, clarifying that it is based on the DFT*.

### Reviewer's report 3

Dr Rob Knight, University of Colorado, Boulder

In this manuscript, the authors compare several methods (autocorrelation, two kinds of Fourier transform, and a hybrid measure) for finding periodicity in DNA sequences. They use as a benchmark the well-established period-10 pattern associated with nucleosome formation. The techniques are benchmarked using data from yeast (using published data from ChIP studies in yeast, which should be enriched in nucleosome-associated DNA), then applied to mammalian genomes. Interestingly, the methods that perform best on simulated and real data are not the same, although both agree that the period-10 pattern dominates. Overall, the techniques chosen seem reasonable and the tests appropriate. The introductory material provides a good overview for non-specialists who may not be familiar with the techniques, although it would perhaps be useful to add some more information about how the methods would be expected to perform based on their formal definitions.

***Author response***: *For the exploratory methods, their expected performance is explained in detail in *[11]. *For confirmatory methods, we make comments in the Methods and Results concerning the theoretical basis for statistical performance. For instance, see ".. deflation of the Hybrid estimates relative to those of the IPDFT is due to the autocorrelation factor.."*.

The discussion and conclusions are relatively sparse. Were other periodicities detected, and if so do any of them have interesting implications? Were the fractions of the genome with dominant period-10 signals unexpectedly high or low? How much did the different methods differ in the whole-genome analysis, and would the biological conclusions change depending on which method was used?

***Author response***: *Other periodicities were certainly evident in the data (see *Figure 5*). As we were focussed on establishing the statistical properties of the techniques rather than a full examination of periodic signals in genomes we did not do exploratory period estimation for the whole-genome analyses. Thus, we cannot comment on the relative significance of period-10 compared to other periods. Regarding the impact of method choice on biological conclusions, our analyses firmly establish that the chosen metric can have a profound impact (see *Table 1*). Specifically, the Autocorrelation and DFT techniques would lead to substantial underestimation of NPS abundance potentially causing the biological significance of these sequence properties to be similarly underestimated. Highlighting the sensitivity of the conclusions to the chosen metric is a major objective of the work*.

The manuscript would also benefit from adding to the conclusions some general discussion about why this problem is important and what tasks can now be tackled with the benchmarking done in this paper.

***Author response***: *We concur with this suggestion and have now modified the manuscript accordingly*.

Overall, the manuscript is a useful contribution to the literature and is suitable for Biology Direct.

## Supplementary Material

Additional file 1 **Significant period 10 regions in mouse chromosomes 1-9**. A bzip (mouse-sig_05-period_10-1-9.bed.bz2) compressed bed format, suitable for visualisation as a track at genome portals. Click here for file

Additional file 2 **Significant period 10 regions in mouse chromosomes 10-Y**. A bzip (mouse-sig_05-period_10-10-Y.bed.bz2) compressed bed format, suitable for visualisation as a track at genome portals. Click here for file

Additional file 3 **Significant period 10 regions in yeast**. A bzip (yeast-sig_05-period_10.bed.bz2) compressed bed format, suitable for visualisation as a track at genome portals. Click here for file

Additional file 4 **A pdf (epps_ying_huttley_supplementary.pdf) showing the SNR calculation**. Click here for file

## References

[B1] Trifonov EN Sussman JL The pitch of chromatin DNA is reflected in its nucleotide sequence Proc Natl Acad Sci USA 1980 77 7 3816 3820 10.1073/pnas.77.7.3816 6933438 PMC349717

[B2] Satchwell SC Drew HR Travers AA Sequence periodicities in chicken nucleosome core DNA J Mol Biol 1986 191 4 659 75 10.1016/0022-2836(86)90452-3 3806678

[B3] Segal E Fondufe-Mittendorf Y Chen L Thastrom A Field Y Moore IK Wang JPZ Widom J A genomic code for nucleosome positioning Nature 2006 442 7104 772 778 10.1038/nature04979 16862119 PMC2623244

[B4] Mavrich TN Jiang C Ioshikhes IP Li X Venters BJ Zanton SJ Tomsho LP Qi J Glaser RL Schuster SC Gilmour DS Albert I Pugh BF Nucleosome organization in the Drosophila genome Nature 2008 453 7193 358 62 10.1038/nature06929 18408708 PMC2735122

[B5] Valouev A Ichikawa J Tonthat T Stuart J Ranade S Peckham H Zeng K Malek JA Costa G McKernan K Sidow A Fire A Johnson SM A high-resolution, nucleosome position map of C. elegans reveals a lack of universal sequence-dictated positioning Genome Res 2008 18 7 1051 63 10.1101/gr.076463.108 18477713 PMC2493394

[B6] Fernandez AG Anderson JN Nucleosome positioning determinants J Mol Biol 2007 371 3 649 68 10.1016/j.jmb.2007.05.090 17586522

[B7] Ponts N Harris EY Prudhomme J Wick I Eckhardt-Ludka C Hicks GR Hardiman G Lonardi S Le Roch KG Nucleosome landscape and control of transcription in the human malaria parasite Genome Res 2010 10.1101/gr.101063.109 PMC2813478 20054063

[B8] Luger K Mäder AW Richmond RK Sargent DF Richmond TJ Crystal structure of the nucleosome core particle at 2.8 A resolution Nature 1997 389 6648 251 60 10.1038/38444 9305837

[B9] Jiang C Pugh BF Nucleosome positioning and gene regulation: advances through genomics Nat Rev Genet 2009 10 161 172 19204718 10.1038/nrg2522 PMC4860946

[B10] Widom J Short-range order in two eukaryotic genomes: relation to chromosome structure J Mol Biol 1996 259 4 579 88 10.1006/jmbi.1996.0341 8683566

[B11] Epps J A hybrid technique for the periodicity characterization of genomic sequence data EURASIP J Bioinform Syst Biol 2009 924601 10.1155/2009/924601 PMC3171443 19365578

[B12] Peng CK Buldyrev SV Goldberger AL Havlin S Sciortino F Simons M Stanley HE Long-range correlations in nucleotide sequences Nature 1992 356 6365 168 70 10.1038/356168a0 1301010

[B13] Li W The study of correlation structures of DNA sequences: a critical review Computers & chemistry 1997 21 4 257 271 10.1109/GENSIPS.2008.4555661 9415988

[B14] Herzel H Trifonov E Weiss O Grosse I Interpreting correlations in biosequences Physica A 1998 249 1-4 449 459 10.1016/S0378-4371(97)00505-0

[B15] Salih F Salih B Trifonov EN Sequence structure of hidden 10.4-base repeat in the nucleosomes of C. elegans J Biomol Struct Dyn 2008 26 3 273 82 18808193 10.1080/07391102.2008.10531241

[B16] Bettecken T Trifonov EN Repertoires of the nucleosome-positioning dinucleotides PLoS One 2009 4 11 e7654 10.1371/journal.pone.0007654 19888331 PMC2765632

[B17] Stewart M McLachlan AD Fourteen actin-binding sites on tropomyosin? Nature 1975 257 5524 331 3 10.1038/257331a0 1161036

[B18] Silverman BD Linsker R A measure of DNA periodicity J Theor Biol 1986 118 3 295 300 10.1016/S0022-5193(86)80060-1 3713213

[B19] Dodin G Vandergheynst P Levoir P Cordier C Marcourt L Fourier and wavelet transform analysis, a tool for visualizing regular patterns in DNA sequences Journal of Theoretical Biology 2000 206 3 323 326 10.1006/jtbi.2000.2127 10988018

[B20] Kumar L Futschik M Herzel H DNA motifs and sequence periodicities In Silico Biol 2006 6 1-2 71 8 16789915

[B21] Epps J Ambikairajah E Akhtar M An integer period DFT for biological sequence processing Genomic Signal Processing and Statistics 2008 1 4

[B22] Sethares WA Staley TW Periodicity transforms IEEE transactions on Signal Processing 1999 47 11 2953 2964 10.1109/78.796431

[B23] Muresan D Parks T Orthogonal, exactly periodic subspace decomposition IEEE Transactions on Signal Processing 2003 51 9 2270 2279 10.1109/TSP.2003.815381

[B24] Arora R Sethares W Bucklew J Latent Periodicities in Genome Sequences Selected Topics in Signal Processing, IEEE Journal of 2008 2 3 332 342

[B25] Berger J Mitra S Astola J Power spectrum analysis for DNA sequences Seventh International Symposium on Signal Processing and Its Applications, 2003. Proceedings 2003 29 32

[B26] Yuan GC Liu JS Genomic sequence is highly predictive of local nucleosome depletion PLoS Comput Biol 2008 4 e13 10.1371/journal.pcbi.0040013 18225943 PMC2211532

[B27] McLachlan AD Stewart M The 14-fold periodicity in [alpha]-tropomyosin and the interaction with actin Journal of Molecular Biology 1976 103 2 271 298 10.1016/0022-2836(76)90313-2 950663

[B28] Chechetkin VR Turygin A Search of hidden periodicities in DNA sequences J Theor Biol 1995 175 4 477 94 10.1006/jtbi.1995.0155 7475085

[B29] Illingworth C Parkes K Snell C Mullineaux P Reynolds C Criteria for confirming sequence periodicity identified by Fourier transform analysis: application to GCR2, a candidate plant GPCR? Biophysical chemistry 2008 133 1-3 28 35 10.1016/j.bpc.2007.11.004 18086512

[B30] Chaley MB Korotkov EV Skryabin KG Method revealing latent periodicity of the nucleotide sequences modified for a case of small samples DNA Res 1999 6 3 153 63 10.1093/dnares/6.3.153 10470846

[B31] Ahdesmäki M Lähdesmäki H Pearson R Huttunen H Yli-Harja O Robust detection of periodic time series measured from biological systems BMC Bioinformatics 2005 6 117 10.1186/1471-2105-6-117 15892890 PMC1168888

[B32] Ptitsyn AA Zvonic S Gimble JM Permutation test for periodicity in short time series data BMC Bioinformatics 2006 7 Suppl 2 S10 10.1186/1471-2105-7-S2-S10 17118131 PMC1683571

[B33] Ying H Epps J Williams R Huttley G Evidence that localized variation in primate sequence divergence arises from an influence of nucleosome placement on DNA repair Mol Biol Evol 2010 27 3 637 49 10.1093/molbev/msp253 19843619 PMC2822288

[B34] Karlin S Campbell AM Mrázek J Comparative DNA analysis across diverse genomes Annu Rev Genet 1998 32 0066-4197 185 225 9928479 10.1146/annurev.genet.32.1.185

[B35] Bernardi G Isochores and the evolutionary genomics of vertebrates Gene 2000 241 3 17 10.1016/S0378-1119(99)00485-0 10607893

[B36] Voss R Evolution of long-range fractal correlations and 1/f noise in DNA base sequences Physical Review Letters 1992 68 25 3805 3808 10.1103/PhysRevLett.68.3805 10045801

[B37] Lee W Tillo D Bray N Morse R Davis R Hughes T Nislow C A high-resolution atlas of nucleosome occupancy in yeast Nat Genet 2007 10.1038/ng2117 17873876

[B38] Rife D Boorstyn R Single tone parameter estimation from discrete-time observations Information Theory, IEEE Transactions on 1974 20 5 591 598 10.1109/TIT.1974.1055282

[B39] Tretter S Estimating the frequency of a noisy sinusoid by linear regression IEEE Transactions on Information Theory 1985 31 6 832 835 10.1109/TIT.1985.1057115

[B40] Fisher RA Tests of significance in harmonic analysis Proc R Soc A 1929 125 796 54 59 10.1098/rspa.1929.0151

[B41] Wichert S Fokianos K Strimmer K Identifying periodically expressed transcripts in microarray time series data Bioinformatics 2004 20 5 20 10.1093/bioinformatics/btg364 14693803

[B42] Raup DM Sepkoski JJ Jr Periodicity of extinctions in the geologic past Proc Natl Acad Sci USA 1984 81 3 801 5 10.1073/pnas.81.3.801 6583680 PMC344925

[B43] Knight RD Freeland SJ Landweber LF A simple model based on mutation and selection explains trends in codon and amino-acid usage and GC composition within and across genomes Genome Biol 2001 2 4 RESEARCH0010 11305938 10.1186/gb-2001-2-4-research0010 PMC31479

