# Supplementary material for: Statistical methods for detecting periodic fragments in DNA sequence data
Source: Biol Direct. 2011 Apr 28;6:21. doi: 10.1186/1745-6150-6-21 (PMC3111405; doi:10.1186/1745-6150-6-21)
Supplement: Additional file 4 — A pdf (epps_ying_huttley_supplementary.pdf) showing the SNR calculation. [file 1745-6150-6-21-S4.PDF]

Appendix A - Epps, Ying, Huttley

SNR Calculation

In this work a frequency domain estimate of the SNR is computed as

$$\frac{\hat{A}^2}{\sigma_w^2} = \frac{\int_0^\pi |X_{perfect}(\theta)|^2 d\theta}{\int_0^\pi ||X(\theta)| - |X_{perfect}(\theta)||^2}, \tag{1}$$

where

$$X_{perfect}(\theta) = X(\hat{\theta}) \sum_{k=1}^{2\pi/\hat{\theta}} \frac{\sin\left(\frac{N(\theta - k\hat{\theta})}{2}\right)}{\sin\left(\frac{\theta - k\hat{\theta}}{2}\right)}, \tag{2}$$

and $\hat{p} = 2\pi/\hat{\theta} \in \mathbb{N}$. A convenient alternative to calculating the sum term in $X_{perfect}(\theta)$ for finite length sequences is to take the discrete Fourier transform of the sequence $\sum_{k=0}^{\lfloor N/\hat{p} \rfloor} \delta[n - k\hat{p} + n_0]$, where n_0 can be set to zero without loss of generality since we are using spectral magnitudes in the SNR calculation.
